# Inequality in urban green space benefits: Combining street greenery and park greenery

**DOI:** 10.1371/journal.pone.0273191

**Published:** 2022-09-19

**Authors:** Chenlu Xue, Cheng Jin, Jing Xu

**Affiliations:** 1 School of Geography, Nanjing Normal University, Nanjing, Jiangsu, China; 2 Jiangsu Center for Collaborative Innovation in Geographical Information Resource Development and Application, Nanjing, Jiangsu, China; 3 Tourism and Social Administration College, Nanjing Xiaozhuang University, Nanjing, Jiangsu, China; Northeastern University (Shenyang China), CHINA

## Abstract

In this paper, we measured the amount of urban green space (UGS), defined here as park greenery and street greenery, in the Guangzhou Beltway region using remote sensing image data and the green view index (GVI) based on human visual images. We also evaluated the benefits of UGS comprehensively considering park greenery and street greenery within the Guangzhou Beltway region. We then calculated the urban green space score (UGSS) by assessing the amount of street greenery and park greenery and then juxtaposing the score with the population distribution of the region. The results show inequities in the spatial distribution of UGSS values within the Guangzhou Beltway region. The benefit score of street greenery is low. The service area of parks can’t cover the whole study area. The comprehensive benefit score of UGS is composed of two parts, the park greenery score and the street greenery score, but the spatial distribution of UGSS values remains uneven. The UGS benefits enjoyed by one-half of the population of the study area are low, and the UGSS values of the more densely populated areas are not high.

## Introduction

People are gradually becoming more conscious of environmental issues and environmental protection. Urban green space (UGS) plays an important role in urban planning and construction [1, 2] for it is an important indicator of quality of life [3, 4]. Urban green space has a positive effect on the urban ecological environment, social and economic development, and residents’ physical and mental health [5, 6]. The most prominent natural by-product of UGS is good air quality [6, 7]. UGS enables the absorption of carbon emissions, increases the supply of oxygen in the air, and lowers PM2.5 levels [8–10]. UGS also plays a positive role in regulating urban microclimate, abating noise, and increasing biodiversity [11, 12]. In addition to protecting the natural and living environment [6], UGS creates an ecological premium that drives up land and house prices [13–15], has a positive effect on regional economic growth [16], and helps to promote social harmony [6]. For residents, UGS provides places of leisure and recreation, optimizes the living and working environment [17], and promotes the frequency of outdoor activities [18, 19], all of which have a positive effect on physical and mental health [5, 20, 21].

How should the quantity and quality of UGS and its equity maps be measured [22–25]? How is the spatial disparity of UGS accessibility? The two basic questions are fundamental for all the UGS research. This paper aims at measuring UGS in comprehensive way, and analyzing the spatial disparity of UGS accessibility.

### Urban green space measurement

UGS is composed of natural forests and artificial green infrastructure, which can be divided into two types, public green space and private green space, according to owners [2, 3]. Most research paid attention to the public UGS which can be entered for residents without restriction as public goods [2].

The question of how to measure the quantity and quality of UGS is of concern for many scholars and is the basis of our research. The most widely used quantitative index for UGS evaluation is normalized difference vegetation index (NDVI) [6]. The NDVI calculates the grid value of UGS images using the ray principle, obtains the green pixels by threshold screening, and calculates the proportion of the green pixels combined with the measuring unit [19, 26, 27]. Satellite remote sensing images provide a bird’s eye perspective of UGS data [27]. Bird’s eye view images of UGS have been used to accurately capture images of large green spaces such as public parks.

The importance of street greenery has been validated in several researches [28–31]. However, street greenery accounts for only a small proportion of the greenery captured by remote sensing satellite images [19] and it is not always possible to identify and capture street greenery accurately or completely using bird’s eye view images [32]. To present the street greenery better, Green View Index (GVI) is based on human visual images which now used most widely measurement tool for street greenery [33, 34]. The GVI is used to express the amount of UGS seen by people from any position in the city [33]. The human visual images and vegetation recognition are now two important aspects affecting the accuracy of GVI. Currently, the most accessible and abundant data sources are panoramic images provided by various online map platforms. Researchers overseas use Google Street View (GSV) to calculate GVI [34, 35], whereas researchers in China use panoramic images of Baidu Maps [36] or Tencent Maps [37]. The methodologies for the recognition and extraction of green vegetation indicators in images are improving continuously. These include the RGB channel comparison method [17, 37], the HSV value calculation method [37, 38], and deep machine learning [39, 40], etc. However, it should be said that all these extraction methods are complicated to use.

### Urban green space accessibility

Accessibility is an important UGS factor with ever-growing implications (e.g., distance, travel time, facilities, etc,) that directly affect whether residents go to the UGS or not. Accessibility analysis is vital for environmental justice research [41]. Most of the accessibility analysis of UGS is based on the remote sensing data whose mainly focus on large area green space such as park greenery.

Combined with the activities or behavior of residents, park accessibility may be defined as the distance from the community to the nearest park, including straight distance or distance along the streets [3, 42, 43]. In addition, green space evaluation research also study park characteristics such as park area, facilities, landscape, and aesthetics. An evaluation study on Shanghai parks by Fan et al. [2] explored two aspects of accessibility—travel distance to physical space using different traffic modes, and the quality of parks with regard to facilities, area, quietness, spaciousness, etc. Jang et al., [44] agreed that UGS accessibility research should not only study the distance to parks, but also the amount of greenery in UGS, the distance to the nearest road, and the topological importance (i.e., traffic flow on the road) involved in access to UGS.

These connotations of accessibility may be then used to evaluate the service capacity of parks with regard to green benefits and/or attractions the park can provide. Studies have also defined accessibility as the attractiveness of park greenery, park area (e.g., size), and the distance from any point to the parks [45, 46]. In evaluating the service capacity of park greenery, characteristics such as park area, distance to the park, and functional scope of the park are the basic elements of the evaluation [4, 47].

However, street greenery accessibility didn’t get enough attention. The analysis about street greenery is still limited in the road space. Ye et al., coupled road accessibility with street GVI [48], which only focused on the road space and didn’t spread the street greenery service beyond the road space.

In summary, the lack of scientific rigor of street greenery in remote sensing data and the space limitation of street greenery research has led to the separation between park greenery and street greenery in UGS studies. Though the use of satellite images to calculate NDVI values in the measurement and evaluation of park greenery seems like an adequate methodology. However, the evaluation of street greenery lacks scientific rigor. A bird’s eye view of street greenery does not capture the same amount of street greenery that people see at eye level [17, 32]. GVI based on human visual images can measure street greenery better than the indices based on remote sensing data. Nevertheless, street greenery accessibility and service capacity analysis require deeper exploration, evaluation research on street greenery has not yet progressed outward linear green space.

Therefore, this paper combines the street greenery and park greenery to measure UGS and explore the spatial disparity in UGS benefits based on accessibility analysis. This paper defines UGS as comprising of both street greenery and public park greenery as they are the spaces most frequently visited by residents without restriction. We analyzed the park greenery using remote sensing data and street greenery using GVI based on human visual images, and construct the Urban Green Space Score (UGSS) to measure and evaluate the green benefits of UGS in densely populated areas of Guangzhou.

## Methodology and material

### Research approach

In this paper, UGS benefits (e.g., street greenery benefits and park greenery benefits) refers to the degree of UGS accessibility for residents can enjoy at any location in city, which is composed of quantity of the UGS and distance to UGS. We constructed the index of urban green space score (UGSS) to capture (a) street greenery benefits calculated as street greenery score (SGS), and (b) park greenery benefits calculated as park greenery score (PGS) and measured and analyzed them separately.

As part of our data gathering methodology, We randomly selected 100 people to conduct an online questionnaire survey separately on November 21st, 2020. The interview includes two questions. One question is to ask the residents the maximum distance they can accept to walk to the parks. The average value the answers is 1.5 kilometer. The another question is to get their opinions of the importance of street greenery and park greenery. We set a scale of 0 to 10 for the interviewees. The smaller the value chosen by the interviewees is, the more important the street greenery is. Conversely, the more important is the park greenery. By calculating the average value of the values selected by interviewees living within the Guangzhou Beltway, the weight scores between street and park green space was obtained. The weight scores were used to sum up the green benefit value. The service efficiency of UGS were obtained by comparing the values of the green benefits with the population distribution of the study area. Specifically, the number of residents in a geographical unit with a specific UGSS value was evaluated. The definition and calculation steps are described in detail below.

### Measures

#### Street greenery score

The street greenery benefit SGS is directly related to the greenery status of the streets. Taking the block surrounded by streets as the calculation unit, the SGS value enjoyed by residents at any given location of the block includes the greenery status of the enclosed streets calculated as GVI and the distance to the streets.

### (1) GVI Calculation

**Fig 1:**
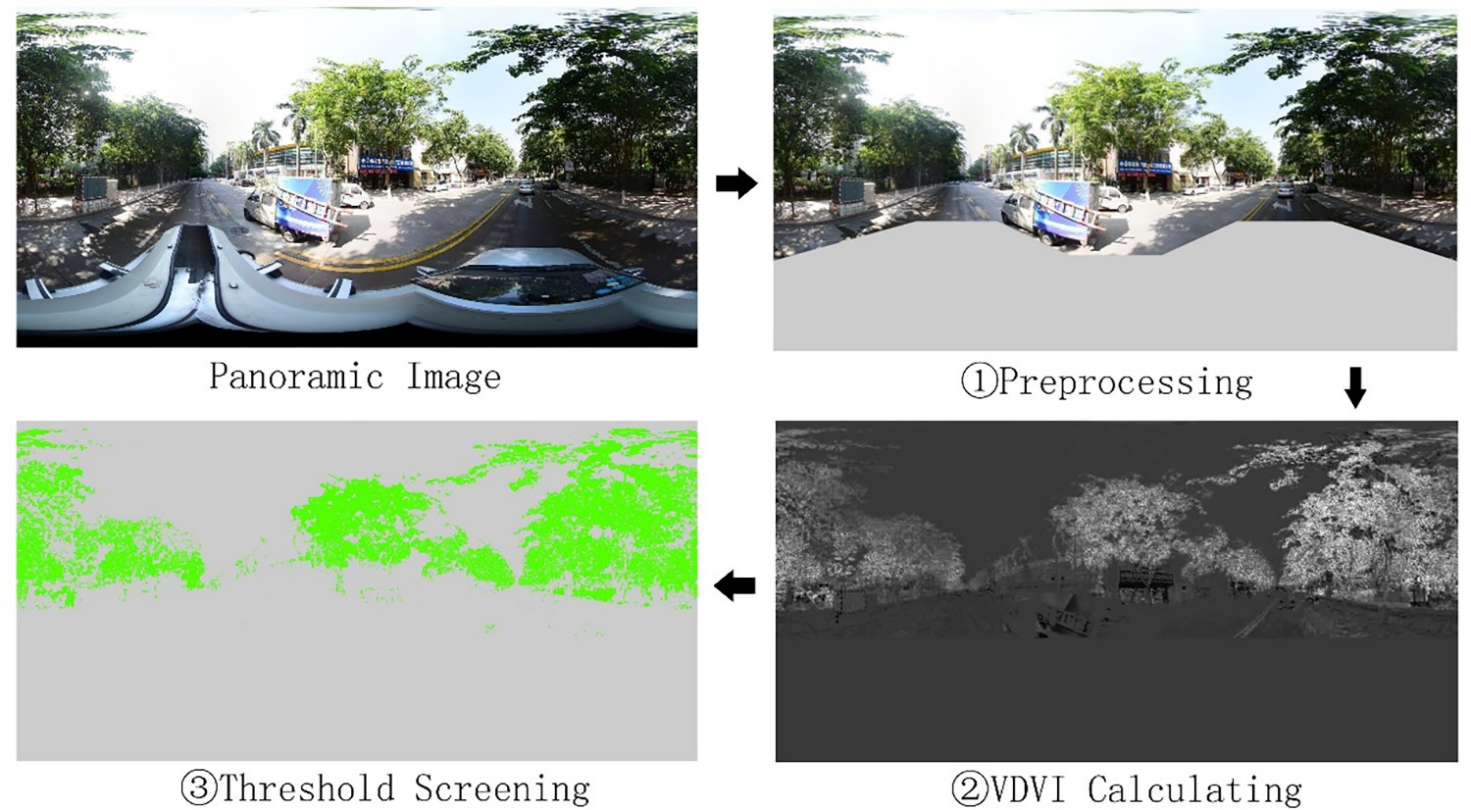
Vegetation extraction in images.

The calculation of GVI at data point i (GVI_i_) was set up as a five-step process. First, the images were pre-processed by masking the body and surrounding pixels of cars in the street view images because car pixels can be misidentified as green pixels. The second step was to calculate the visible-band difference vegetation index values [49] of the pre-processed images and form grayscale images. The third step involved identifying the threshold values. After several experiments on the calculated VDVI values of the images, 0.1 was selected as the threshold value. Only pixels with a value greater than 0.1 were recognized as green pixels. The fourth step was to calculate the GVI values of the four images. The ratio of the number of extracted green pixels to the total number of pixels in the image provided the GVI value of each image. This step was repeated for all four images (Fig 1) at each location point. Finally, the average proportion of green pixels in the four images was calculated and represented the GVI value of the panoramic location point.


$$GVI_{i}=\frac{\sum_{j=4}^{4}\mathrm{GreenPixel}\;si_{ij}}{\sum_{j=4}^{4}\mathrm{TotalPixel}\;s_{ij}}.$$ (1)

In Formula (1), *i* is the serial number of data points, and *j* represents the four directions: east, south, west, and north.

### (2) SGS Calculation

Based on photos of the street network acquired by panoramic images, we formed the blocks. The sum of GVI values of the enclosed streets of each block B_i_ was calculated with the area S_bi_ and perimeter D_bi_. The area and shape of the enclosed block were not uniform. The distance from the center of each block to the enclosed street was expressed, approximately, by the ratio of the perimeter to the area. We divided the sum of GVI values by the distance from the central point to the enclosed streets and standardized it to get the SGS value of the block.


$$SGS_{i}=\arctan(\sum_{bi}GVI*(D_{bi}/S_{bi})).$$ (2)

In Formula (2), *i* is the serial number of block B.

#### Park greenery score

The benefit value of park greenery was calculated as PGS based on the park area and the distance to the park. In principle, the value is higher when the area of the park is larger and the distance to the park is closer to home. In this study, we found that the PGS value of each park was inversely proportional to the distance to the park and directly proportional to the area of the park. As per the interview responses, the maximum distance residents walk to the park is 1.5 km. Therefore, a PGS value exceeding 1.5 km to the park was set to 0, and the PGS value inside the park took the value of the park edge. The PGS value grids of all the parks were laid out in a mosaic and the larger value was taken. After normalization, the benefit value distribution of the parks was obtained.

The specific process was set out as follows: First, we calculated the distance from each location point to a single park P_i_. The cost matrix was generated according to the water system and street network. The value of the water area was set to null, and the value of the street and other areas was set to 1. In the case of walking, the cost of passing through the street network was considered to be the same with it of the other land use. The cumulative cost was obtained using the ArcGIS cost distance tool. We considered that the grid value was the distance L_pi_ to the park P_i_ combined with the grid size. Second, we calculated the PGS values for a single park P_i_. A 1.5 km buffer was formed around the park P_i_. The PGS values for the area outside the park but within the buffer area were calculated by converting the buffer area into a grid form with the value of the area S_pi_ of the park i divided by L_pi_. The PGS values inside the park were equal to the PGS values on the park edge, that is, the area was divided by the pixel side length X. The values outside the park but within the 1.5 km buffer and the values inside the park were merged and normalized to get the PGS_i_ of the park P_i_. The final step was to lay out the PGS values grids of all parks into the new grid and combine it with the scope of the study area. The PGS values outside the park’s 1.5 km buffer were set to 0. The overall PGS values of parks obtained were:

$$PGS_{i}=\arctan[\left(S_{pi}/L_{pi})\cup (S_{pi}/X\right)].$$ (3)

In Formula (3), *i* indicates the serial number of the park.

## Urban green space score

### (1) UGSS Calculation

Interviewees’ responses to questions asking them to evaluate the importance of park greenery and street greenery were similar for the most part. The importance ratio of park greenery and street greenery was 13:12. The weight of park greenery was set to 0.52, and the weight of street greenery was set to 0.48. The overall green benefit value UGSS was calculated by a weighted summation as follows:

UGSS=0.48*SGS+0.52*PGS (4)

### (2) Combined with Population

The UGSS service efficiency were obtained by combining UGSS with the classification of the population distribution in the study area. Areas with 10 or more people per square meter were designated as high population density area "H." Areas with fewer than 10 people per square meter were designated low population density area "L."

Comparisons of high and low UGSS distribution were based on the following definitions: Areas with a UGSS value greater than or equal to 0.5 were designated high-value area "H," indicating that the urban comprehensive greenery status is better in these high-value areas. Areas with a value less than 0.5 and greater than or equal to 0.25 were designated the middle area "M," indicating that the urban comprehensive green benefit is neutral in these areas. Areas with a value less than 0.25 were designated the lower area "L," indicating that the urban comprehensive green benefit is poor in these areas. Areas with higher population density but low UGSS value were also identified.

### Study area and data

#### Study area

Guangzhou, located in South China, is the capital of Guangdong Province and one of the largest cities in South China. In 2019, the resident population was 15.31 million. In 2018, the built-up area covered an area of 1, 324.17 square kilometers, with a green coverage area of 602.50 square kilometers, and a green coverage rate of 45.50%. The Pearl River flows through the city and is an important development axis for Guangzhou. Guangzhou is located in the subtropical coastal area and belongs in the marine subtropical monsoon climate zone. Its vegetation is characterized by evergreen broad-leaved forests that vary little throughout the seasons. Therefore, any seasonal variation in image capture will have a minimal impact on the degree of vegetation captured.

**Fig 2:**
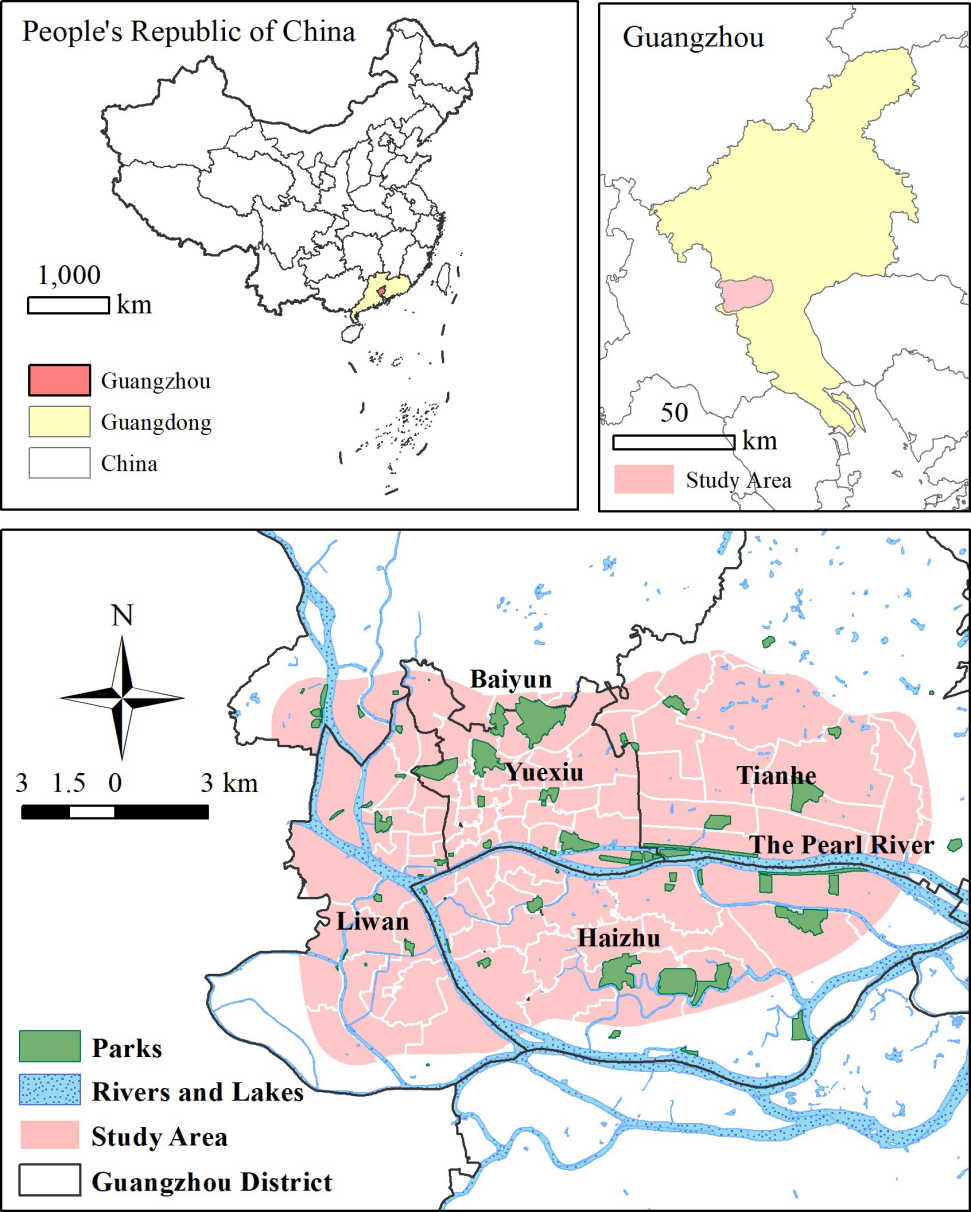
Study area.

Approximately 35% of the total population of Guangzhou is concentrated within the Guangzhou Beltway (according to World pop 2020), an area that represents about 3% of the total area of Guangzhou. This area is the core of the built area and is also the political, cultural, residential, and economic center of Guangzhou. This paper focus on the evaluation of UGS in densely populated urban areas, try to solve the equal provision of UGS benefits for residents under more densely population and socio-economic activities distribution. We selected the Guangzhou Beltway as the boundary of the study area (Fig 2).

Nearly 80% of the study area is built area. Residential area account for 40% of built area in the study area, which accounts for just 20% of the total built area in Guangzhou. Administrative, educational, medical, sports and culture area accounts for the about 24% of built area in the study area, which is of only 13% in Guangzhou built area. Business and commercial area accounts for 27% while industrial area is 17% of the built area in the study area. Transportation area covers about 12% while some parks accounts for 1% of built area in the study area. The study area covers Liwan District, Yuexiu District, Tianhe District, the south of Baiyun District, and the north of Haizhu District. It is composed of 65 complete towns or streets (hereafter referred to as towns) with some areas containing 22 towns. The most important water body in the study area is the Pearl River and its west channel and back channel. Many smaller rivers and inner lakes and pits are scattered in the study area.

#### Data source

Baidu Maps panoramic static images were used to calculate the GVI and SGS scores. According to the JavaScript API interface provided by Baidu Maps, the program requires a panoramic data acquisition service and a panoramic static images service to complete the acquisition of Baidu Maps panoramic image data. A data point every 20m was required in order to include the images and the information for image ID and the WGS84 coordinate position. The 360° field of view angle was divided into four sections corresponding to north, south, east, and west such that four images were obtained at each data point. Each image included a vertical angle view from –45° to 45°. There were 318,496 data points and 1,273,984 panoramic static images of Baidu Maps in the study area.

Based on the geographic registration of the Baidu Maps images and point of interest (POI) data, we drew parks in ArcGIS SHP. format to obtain data on park name, category, area, and location. We found that the maximum distance residents walked to the park was 1.5 km. The acquisition scope of the parks included the 1.5 km buffer zone of the study area. We used the Guangzhou Parks List (2016) and the search results on Baidu Maps to obtain the data. As a green space service for residents, parks data were obtained only for parks that were free of charge—a total of 68 parks. Baiyunshan Park in Guangzhou was not included. The distribution of parks can be seen in Fig 2. and the specific information are listed in Table 1.

**Table 1:** The information of public parks in Guangzhou.

Name	Types	District	Area/ha	Name	Types	District	Area/ha
Structure Park	Special	Baiyun	36.28	Xijiao Shengtai Park	Community	Liwan	2.39
Binjiang Park	Community	Baiyun	12.27	Huadihe Park	Community	Liwan	1.22
MountainTop Park	Community	Baiyun	10.30	Shamian Park	Community	Liwan	0.83
Jinsha Greenheart park	Community	Baiyun	6.60	Mini Physical Park	Community	Liwan	0.58
Kite Park	Community	Baiyun	6.43	Nanjiaocun Park	Community	Liwan	0.46
Luochong Park	Community	Baiyun	3.49	Dongdun Park	Community	Liwan	0.37
Tongde Park	Community	Baiyun	1.87	Tianhe Park	Comprehensive	Tianhe	68.06
Chengxi Park	Community	Baiyun	1.05	Pearlriver Park	Comprehensive	Tianhe	29.82
Fengxiang Park	Community	Baiyun	0.16	Yanling Park	Community	Tianhe	23.97
Haizhu Lake Park	Comprehensive	Haizhu	93.10	Near river park	Community	Tianhe	22.25
Shangchong Guoshu Park	Special	Haizhu	89.14	Haixinsha	Square or similar	Tianhe	17.93
Dawei Park	Community	Haizhu	85.05	Yangtao Park	Community	Tianhe	13.16
Longtan Guoshu Park	Comprehensive	Haizhu	57.16	Near river park 1	Community	Tianhe	13.11
Caochongen Structure Park	Special	Haizhu	29.82	Changban Park	Community	Tianhe	8.91
Qinshui Park	Community	Haizhu	28.51	19 monetary Park	Special	Tianhe	5.15
Huizhan Park	Community	Haizhu	22.70	Qiqiao Park	Community	Tianhe	1.86
Pazhou Tower	Community	Haizhu	18.80	Xiwei Park	Community	Tianhe	1.23
Xiaogang Park	Comprehensive	Haizhu	16.71	Luhu	Comprehensive	Yuexiu	148.21
Zhuangtou Park	Comprehensive	Haizhu	7.55	Yuexiu Park	Comprehensive	Yuexiu	70.65
Qiaotou Park	Community	Haizhu	6.22	Liuhuahu Park	Comprehensive	Yuexiu	54.79
Modiesha Park	Community	Haizhu	5.70	Dongshanhu Park	Comprehensive	Yuexiu	47.26
Chigang Tower	Comprehensive	Haizhu	5.61	Guangzhou uprising martyr cemetery	Special	Yuexiu	18.57
GZ Tower Squre	Square or similar	Haizhu	5.60	GZ Development Park	Community	Yuexiu	14.71
Haizhu Children’s Park	Special	Haizhu	5.50	Chuanqi Park	Community	Yuexiu	9.29
Zhoutouju Park	Community	Haizhu	2.92	Hongcheng Prak	Community	Yuexiu	7.56
GZ Volunteer’s Park	Community	Haizhu	1.93	People’s Park	Comprehensive	Yuexiu	6.62
Haiyin Park	Community	Haizhu	0.98	Haiyin Square	Square or similar	Yuexiu	5.19
Liwanhu Park	Comprehensive	Liwan	22.84	Dongfeng Park	Comprehensive	Yuexiu	4.81
Liwan Children’s Park	Special	Liwan	8.82	Ershadao Park	Special	Yuexiu	4.35
Zuiguan Park	Community	Liwan	5.28	Ersha Art Park	Community	Yuexiu	2.46
GZ Cultural Park	Community	Liwan	4.73	Haiyin Square	Square or similar	Yuexiu	2.28
Zengbu Park	Community	Liwan	3.91	Diwang Square	Square or similar	Yuexiu	1.96
Qingnian Park	Community	Liwan	3.39	Dashatou	Square or similar	Yuexiu	1.63
Shuangqiao Park	Community	Liwan	3.38	GZ Yuexiu Children’s Park	Special	Yuexiu	1.50

Population distribution data from the Worldpop (https://www.worldpop.org/) open-source service were downloaded in August 2020 in TIF format. The grid size was 100m*100m, and the grid value was the number of people in each grid.

The water surface and street network data in this study came from OpenStreetMap, an open-source service (https://download.geofabrik.de/asia/china-latest.osm.pbf). The water polygon element SHP file and the Guangzhou street network polyline element SHP file were downloaded in July 2020 from OpenStreetMap.

## Results

### Street greenery benefits

#### Distribution of GVI value points

**Fig 3:**
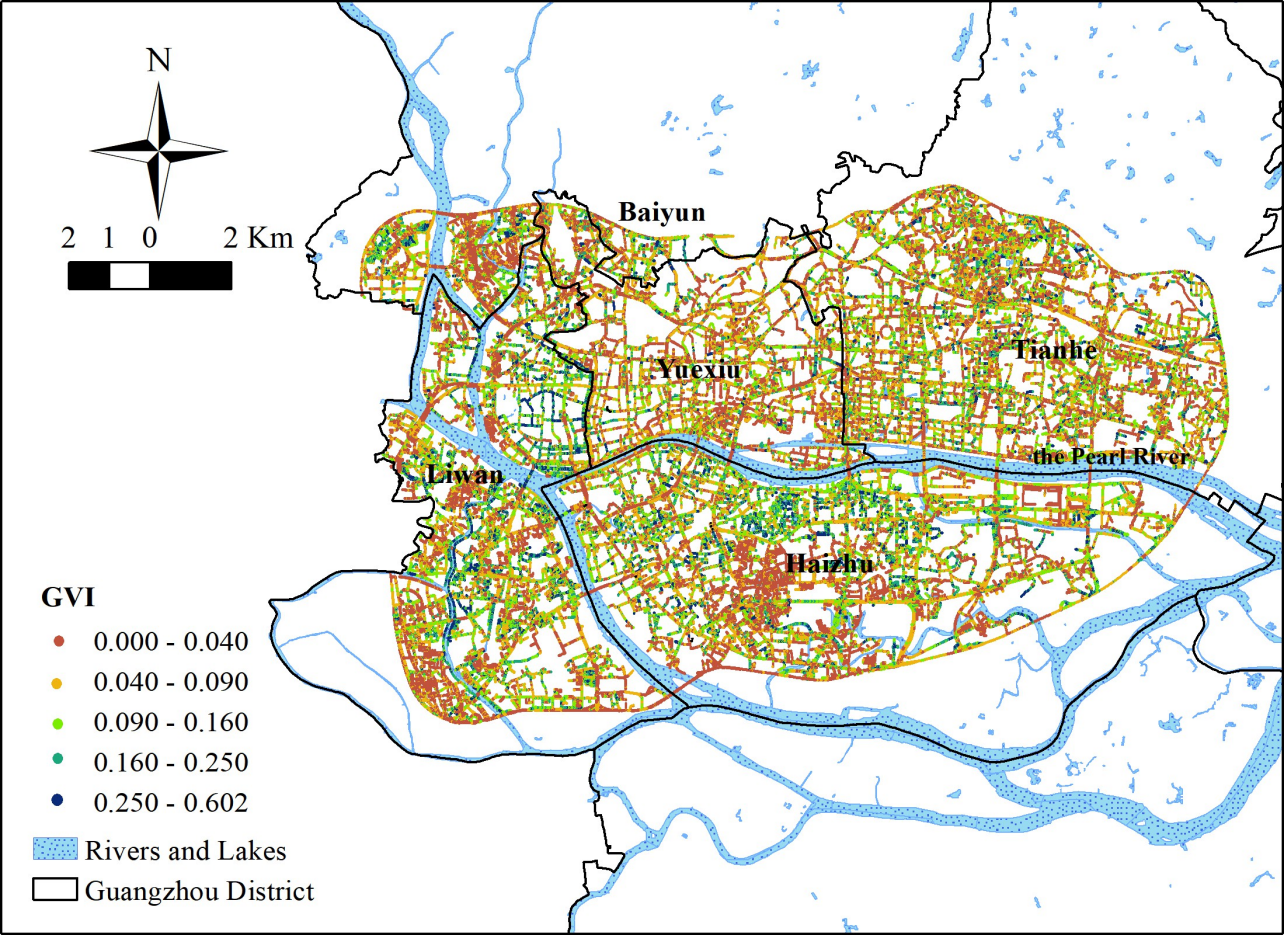
Distribution of GVI points in the study area.

A total of 318,496 data points were generated in the study area (Fig 3), with a maximum of 0.671 in Liwan District and a minimum of 0. The average value was 0.08 and the standard deviation was 0.08. The overall GVI values of the study area were low, less than 0.04. The street greening situation was poor, and high value distributions were few. The areas with higher GVI values were distributed mainly in the west of the study area (Longjin Town, Fengyuan Town, Hualin Town, Lingnan Town, and Shamian Town in Liwan District) and along the Huadi River in the southwest of the study area. The GVI values along the river in the middle and north of Haizhu District were also higher and the street greenery in these areas were also good.

#### Inequality in SGS results

**Fig 4:**
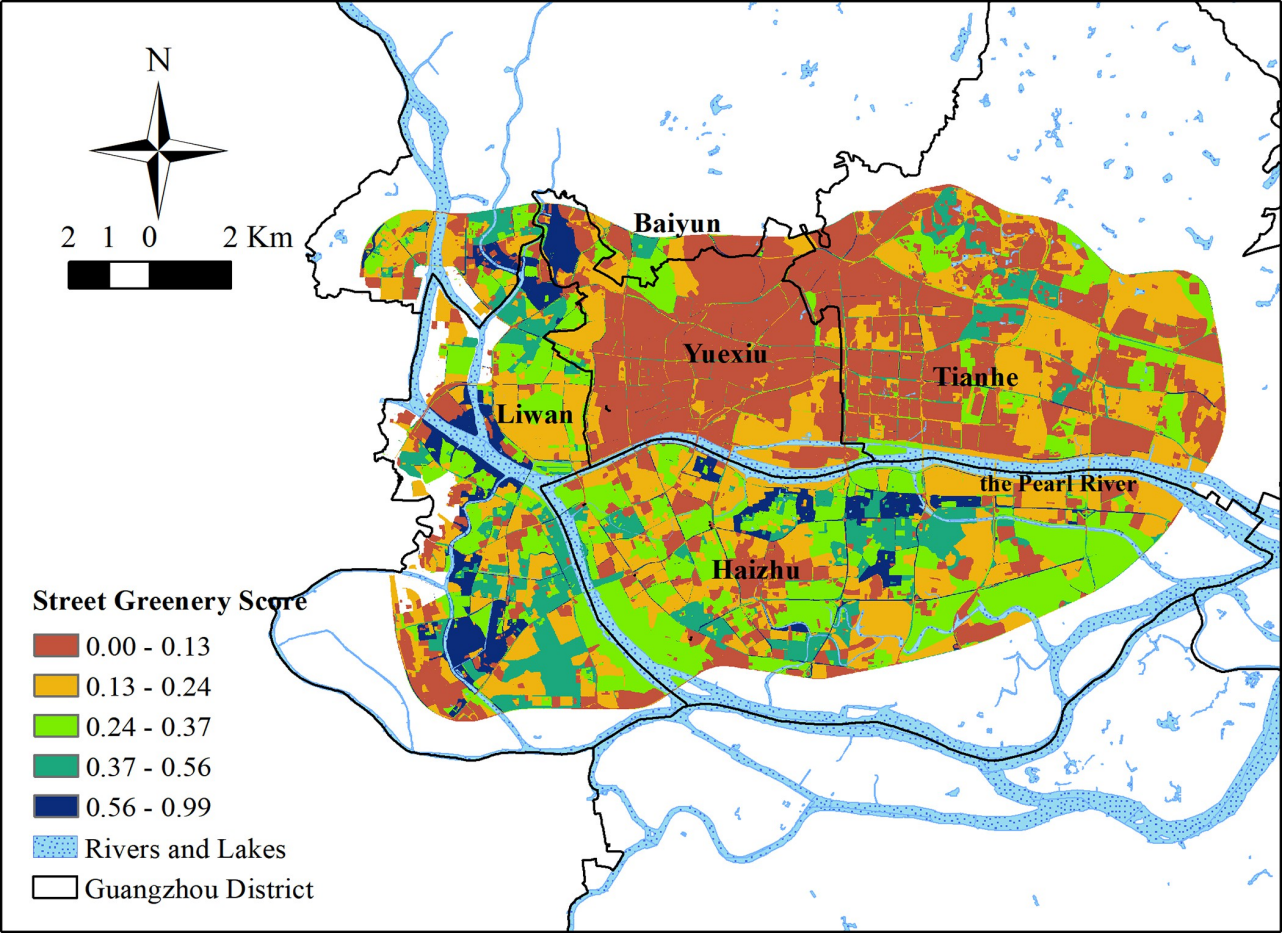
The SGS results in the study area.

The SGS values of the blocks were obtained by combining the GVI values with the area and perimeter of the blocks (Fig 4). The average value was 0.223. The minimum value was 0.002, and the maximum value was 0.985. The standard deviation was 0.164. The area where the SGS values were less than 0.1 accounted for 25% of the total area of the study area; the area with an SGS value less than 0.2 accounted for more than half of the study area. The SGS values of more than 90% of the study area were less than 0.5. Concerning distribution, the areas with high SGS values were similar to areas with high GVI values and included areas in the middle and north of Haizhu District in the west of the study area. There are also some differences with GVI distribution. The SGS values in the south of Baiyun District were higher than the SGS values in Fengyuan Town in Liwan District. The distribution of SGS values was relatively broken and scattered. The SGS values of Tianhe District and the eastern part of Yuexiu District were concentrated but low—most were less than 0.13. The SGS values of streets were higher than that of blocks. This could be because the area of street land is smaller. Generally, the SGS values in Guangzhou were found to be on the low side.

Additionally, due to the inconsistent road grades obtained from Baidu Maps’ panoramic images, the sizes of the enclosed blocks differed. Some high-value areas such as the streets of Xingang may have had higher SGS scores because the panoramic images were of residential roads in the community (which have more greenery) and because the enclosed land area is smaller. Since most of the panoramic images of Tianhe District were of main streets in the larger area (which have less greenery), larger areas had SGS lower scores.

### Park greenery benefits

#### Distribution of parks

A total of 68 free parks were studied—14 comprehensive parks, 9 special parks, 39 community parks, 6 squares or similar, smattering of smaller parks. These parks are distributed mainly in Yuexiu District and Tianhe District and along the Pearl River. The larger parks, are located near constructed lakes in the city or near mountains. The largest park is the comprehensive Luhu Park (1.48 square kilometers) in the north of Yuexiu District, followed by Haizhu Lake Park (0.93 square kilometers), and ShangChong Guoshu Park (0.89 square kilometers) in the south of the Haizhu District. A total of 60 parks measure less than 0.05 square kilometers each, 49 parks of which measure less than 0.02 square kilometers each. Thus there is considerable variation in the size of the parks.

#### Inequality in PGS results

**Fig 5:**
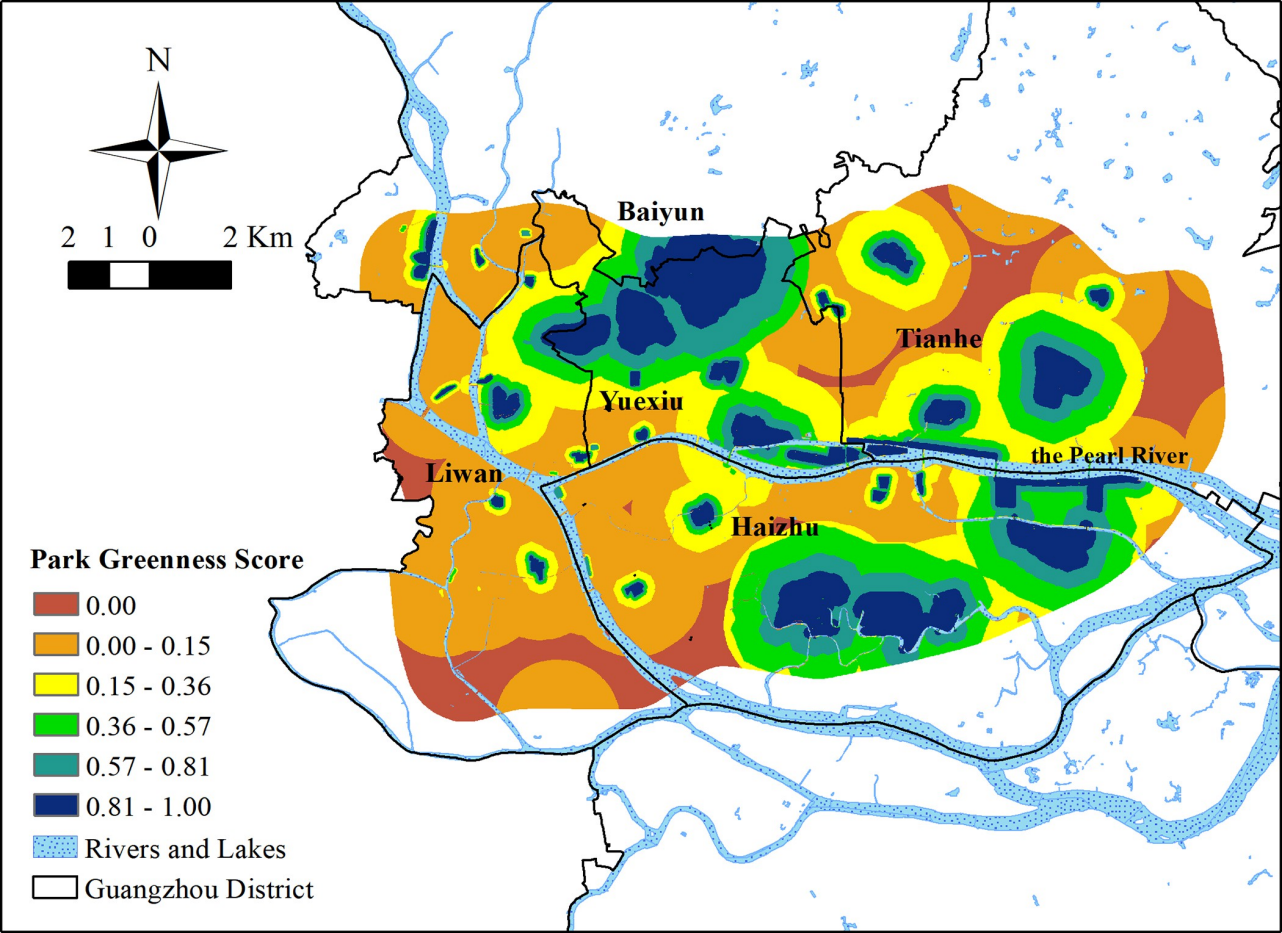
The PGS results in the study area.

The overall average PGS value in the study area was 0.305 (Fig 5). The minimum value was 0 and the maximum value was 0.996. The standard deviation was 0.311. The overall PGS value was higher than the overall SGS value. However, areas with a PGS value of 0 accounted for more than 10% of the study area indicating that not all areas in the Guangzhou Beltway are located within the 1.5 km walking service area of parks. Park green score values of more than 50% of the area were less than 0.2, and areas with a PGS value of more than 0.8 accounted for more than 10% of the study area. Concerning the distribution of parks, areas with higher PGS values were located mainly around large parks in Yuexiu District, the south of Haizhu District, Ersha Island (the island in the Pearl River, to the north of Haizhu District), and the eastern part of the study area. Due to the wide disparity in park areas, most parks with an area of less than 0.02 square kilometers were included within the 1.5 km buffer of larger parks. In the southwest part of the research area, the overall PGS values were lower because of the greater number of small parks.

### Urban green space benefits

**Fig 6:**
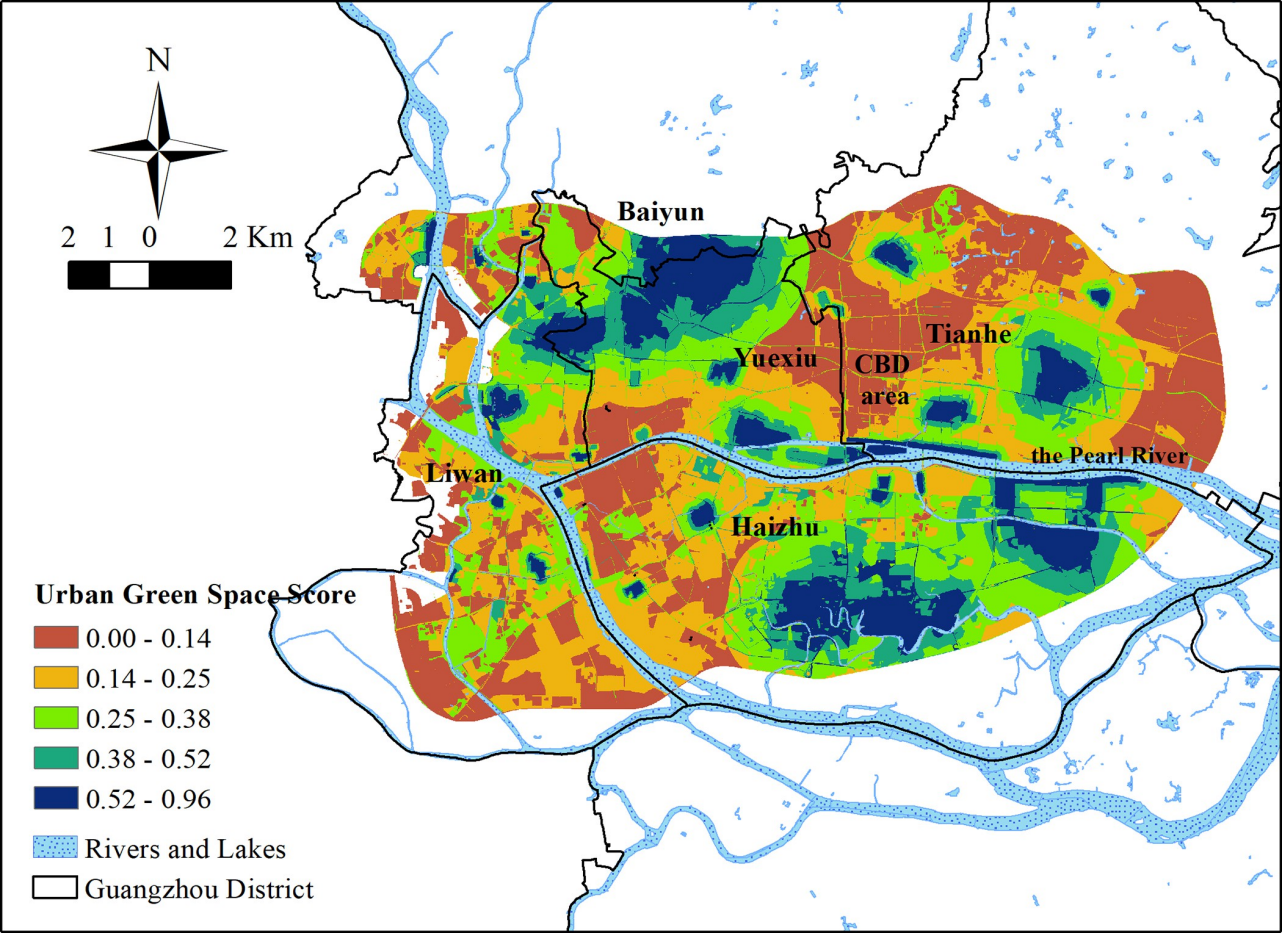
The spatial distribution of UGSS.

**Fig 7:**
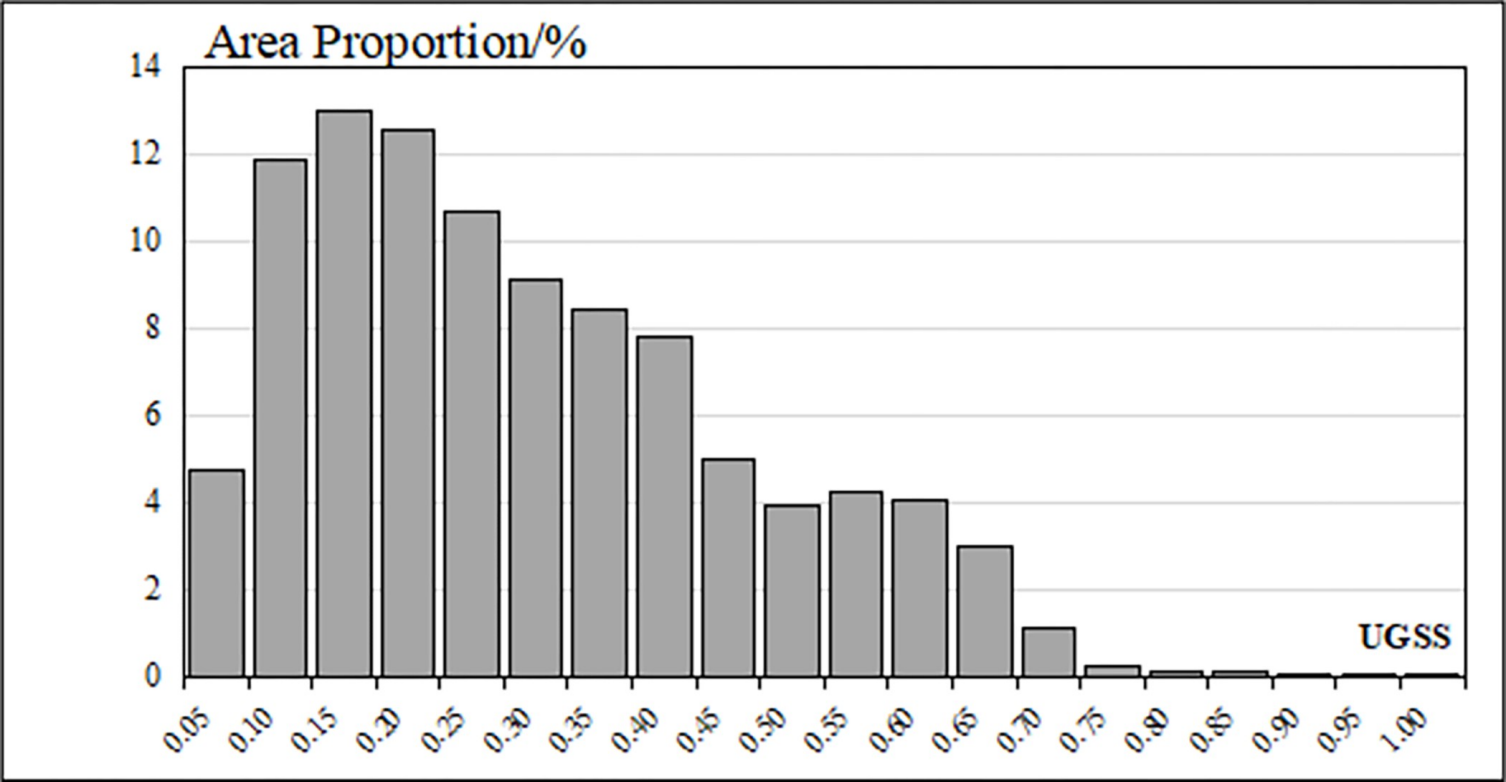
The area distribution of UGSS values.

Concerning the responses to the interviews, the SGS values and the PGS values were weighted and summed up to get the UGSS values (Fig 6). The average UGSS value in the study area was 0.270; the highest value was 0.953; the lowest value was 0.0005. The standard deviation was 0.169. The overall UGSS value was low (Fig 7). One-third of the study area scored lower than 0.15, and half of the study area scored less than 0.25. Areas with UGSS values lower than 0.5 accounted for nearly 90% of the study area. Areas with UGSS values higher than 0.65 accounted for less than 5% of the total area. The area distribution of UGSS values was similar to that of SGS values.

Concerning spatial distribution, the high UGSS value distribution presents as a circular structure around large parks. High SGS values make up for the deficiency of some low PGS value areas; low SGS values lower high PGS values.

However, the inequality in UGSS values is clear. High UGSS value areas are distributed in the north of Yuexiu District, the south of Haizhu District, and the middle section of Tianhe District. The greenery status of the southwest part of the study area was better than in the PGS value distribution map. At present, the UGSS value was found to be lower in the central business district (CBD) area of Guangzhou. Only the Pearl River Park in Xian Village Street scored higher than 0.14.

### Comparison of UGSS results with population distribution

**Fig 8:**
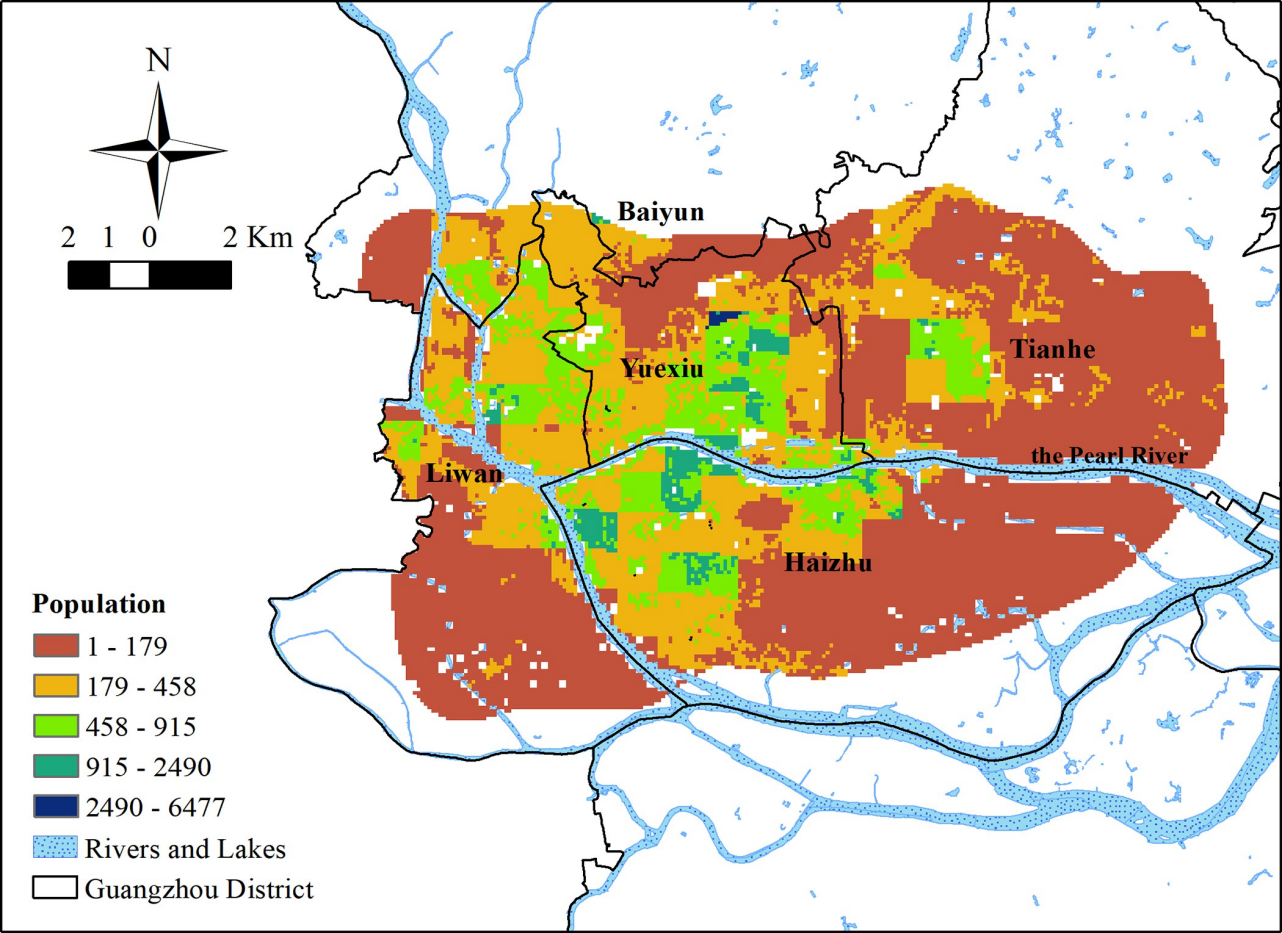
The population distribution in 100m*100m metrics in the study area.

According to the population distribution map of the study area (Fig 8), more than 88% of the study area has a population density of fewer than 5 people per square meter. Areas with more than four to five people per square meter are distributed mainly in the north bank of the Pearl River in Liwan District, Yuexiu District, east of Tianhe District, and in the west and central-north parts of Haizhu District. A higher population density is distributed along the Pearl River and its waterways. The highest density of population is distributed in the south of Dengfeng Town, in the southeast area outside Luhu Park which is dark blue in the Fig 8.

**Fig 9:**
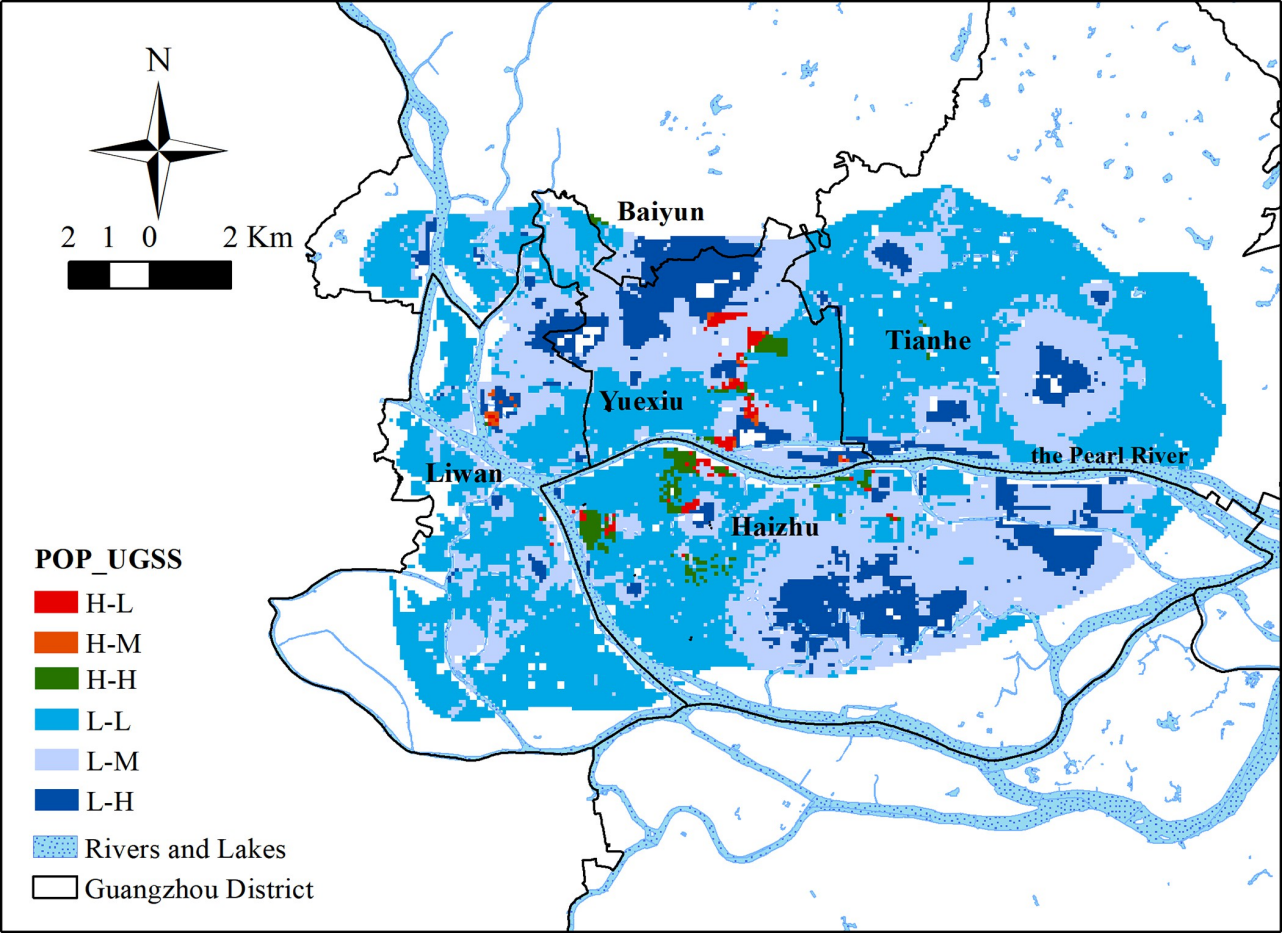
The Spatial distribution of the UGSS service efficiency.

**Fig 10:**
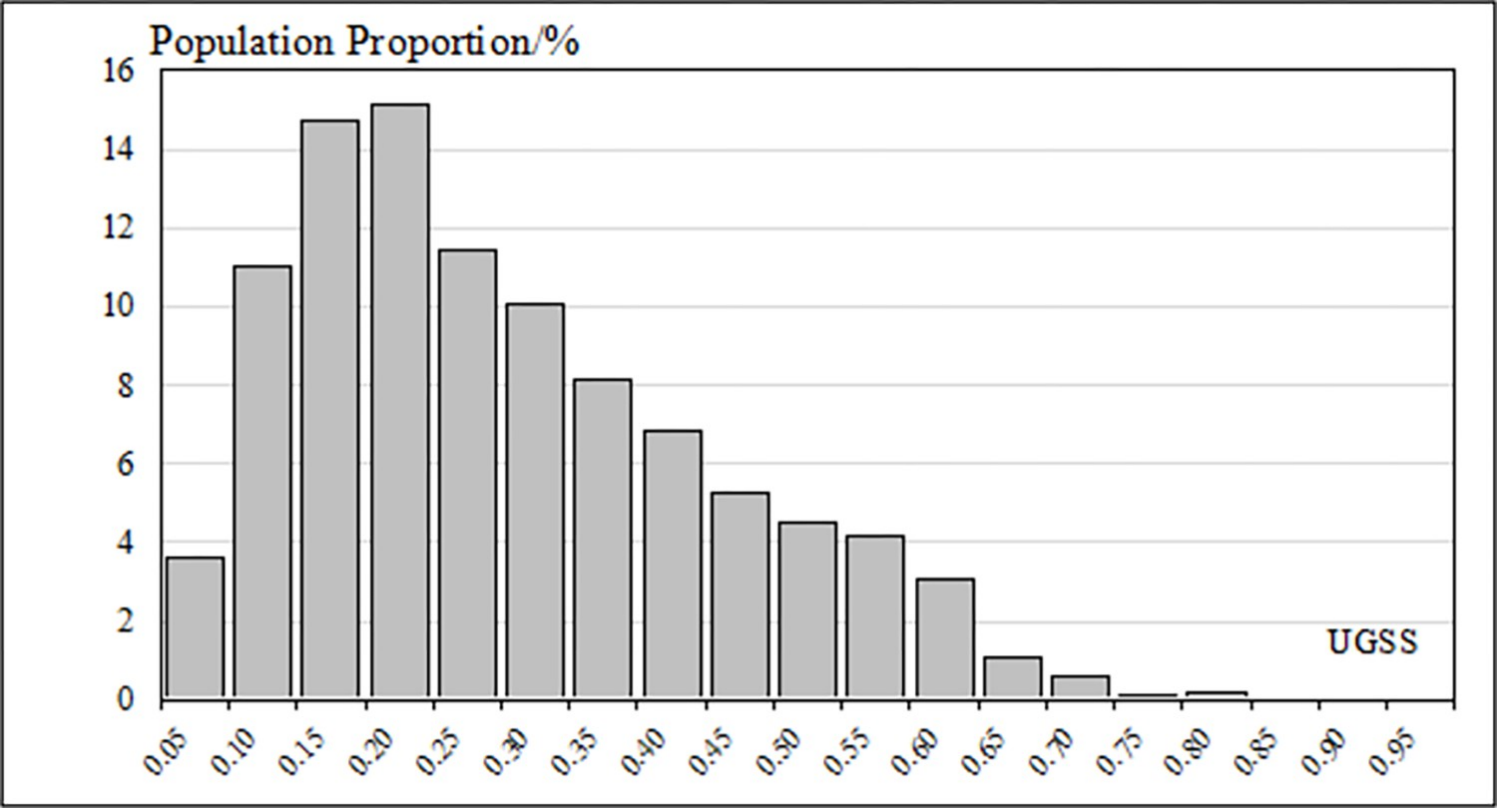
The distribution of population in UGSS results.

The comparison chart (Fig 9) of UGSS values and population and the histogram (Fig 10) of population distribution show the connection of UGSS results to the population distribution within the study area. The actual service efficiency of UGSS distribution, including spatial and population distribution, is uneven for residents. Most of the areas where UGSS values are higher than 0.5 are located near large parks in subsidiary residential areas with a low population density of fewer than 10 people per square meter.

Areas with a population density of more than 10 people per square meter with a UGSS value of less than 0.5 do exist and account for about 1.91% of the study area. This area has about 620,000 people and accounts for about 11.6% of the population in the study area (Worldpop 2020). It is distributed across parts of Longfeng Town, Changgang Town, Binjiang Town, and Chigang Town in Haizhu District, and Huanghuagang Town in the northeast of Yuexiu District, which present red in Fig 9. Only 0.14% of the area overlaps with areas of high population density and high UGSS values. This overlapping section includes over 50,000 people and accounts for about 1% of the population in the study area.

The histogram of UGSS values and population (Fig 10) shows that areas with lower UGSS values had a larger population. More than half of the population in the study area enjoyed green space benefits with scores of less than 0.25. Only about 9% of the population in the study area enjoyed green space benefits with scores of more than 0.5.

## Discussion

### Policy implication

The UGSS equity map includes park greenery benefits analysis and street greenery benefits analysis, based on overlooking data and human visual images respectively. Different from the per capita green space area statistics or the proportion of green space under the administrative unit, by combined with the population distribution, this paper presents the mismatch between UGS benefits supply and demand directly. In the further construction and planning of green space, the results directly show the uneven distribution of UGS benefits in the center of Guangzhou. We can deeply analyze the contribution of street greenery and park greenery situation in the areas with high UGSS value, as well as the construction ideas, and optimize the areas with low UGSS value and high population density according to local conditions.

The overall problems presented in the UGSS distribution are as follows. High UGSS value areas had high PGS values whereas the SGS values were generally low, indicating the requirement to improve street greenery. As the study area is already a fairly built-up area, constructing large green infrastructure is not a feasible idea. More detailed planning (e.g., minor adjustment in greenery) is required. Pocket parks which refers to smaller green space can be an effective way to develop a UGS system. Concerning the construction of parks in the study area, the planning document Guangzhou Urban Green Space System Planning 2020–2035, states that large parks will be maintained and/or rebuilt to adjust the landscape. Bearing in mind the five-minute pedestrian-scale neighborhood in the community as a unit, the construction of a pocket park with an area of less than 0.01 square kilometers could improve the UGSS value and improve UGS benefits and residents’ environmental quality of life.

### Improvement of the UGSS index

As described earlier, previous evaluations of UGS have studied street greenery and park greenery as separate entities. In this paper, we studied street greenery and park greenery as related entities. However, our definition of UGS and its benefits and the methods used for analyzing UGSS are relatively simple. Much more will need to be done to improve the accuracy and generalizability of this index.

How to better combine park greenery based on remote sensing data and street greenery based on human visual data is the most necessary question. The weight scores of street greenery benefit and park greenery benefit are also worthy of further exploration. The results of the interviews indicated that on average, interviewees’ responses were similar. However, individuals did have different perceptions of the relationship between street greenery and park greenery and thus evaluated them differently. For example, the demand for parks by the elderly was higher than demands for parks by the young; the weighted score of the demand for parks by the elderly was higher than the amount of street greenery available to them. Given these findings, subdivided UGS benefits equity maps can convey detailed spatial disparity.

As for the accuracy of the two indicators. On one hand, to calculate the green benefit value of street greenery SGS, we used the GVI value and the block as the unit to be measured. The method of extracting green pixels from the panoramic images is also constantly improving. The accuracy (e.g., time sensitivity and photographing simultaneity of the image) of the Baidu Maps’ panoramic static images will affect the GVI value.

The growth period of plants also has a direct impact on the proportion of greenery visible in the panoramic images and at eye level. Images taken earlier in the planting season and not updated will make for lower GVI values. Meanwhile, the different road grades in the acquisition of Baidu Maps’ panoramic static images can also affect the accuracy and objectivity of the SGS value. The panoramic images of the branch roads between houses can be obtained in some areas. If the width of the road is narrow, the greenery abundant, and the land area formed by low-grade roads is small, the SGS value will be high. In some areas, the lowest road grade obtainable by panoramic images is that of wide main streets. Though the crowns of trees have dense foliage and tree sweaters are wrapped around tree trunks, the proportion of green pixels in the image will be low due to the width of the roads and the GVI value will also be low. If a higher road grade surrounds a large block, the ratio of the perimeter to the area will be smaller and the SGS value will be on the low side.

On the other hand, to calculate the PGS of park greenery benefit value, we considered the ratio of the park area to the distance covered by walking. The calculation of PGS value is accurate for this study area but could be less so for other cities or regions where park areas may be different. Therefore, the park area as a numerical index can also be improved combined with people’s use of parks of different sizes and attitudes to the facilities [20]. In terms of distance, we defined the maximum walking distance to be 1.5 km based on interviewees’ responses. Combined with park size and transportation mode, people’s willingness to go out varies and is based on the available transportation mode and the amenities offered by the service areas of the different types of parks. The cost matrix formed by different transportation modes and land use is also different [45]. Therefore, the analysis and calculation of PGS values with regard to the division of park grade, the definition of the service area and traffic mode, and land use can be more valid and generalizable in other regions.

### Combination of subjective and objective evaluation of UGS

A key question in evaluation research is how to integrate the performance of the objective world with people’s subjective understanding of the world [50]. The evaluation of UGS includes the use of quantitative tools to measure indicators such as the amount of greenery in a given area, facilities in green areas, distance to green areas, and residents’ subjective perceptions of greenery, including aesthetic perceptions. Quantitative evaluation methods produce objectively measurable results, whereas qualitative evaluation methods produce descriptive data that are more subjective. While the tools for capturing both kinds of data can be fine-tuned for ever greater precision, the data gathered from qualitative evaluation methods (e.g., responses to interview or survey questions) are more open to interpretation than numbers. However, the results of both kinds of evaluation tools can support each other. For example, the UGSS index used quantitative methods to measure street greenery benefits and park greenery benefits. However, when comparing these quantitative evaluation results with qualitative evaluation results (e.g., residents’ responses in the interviews), the latter also had a bearing on the research goals of this study. Thus, objective measurements can be combined with people’s subjective evaluation to construct a quantitative and qualitative UGSS evaluation index.

However, improvements in UGS and resultant benefits cannot fully beautify and improve residents’ living environment as per their aesthetic needs. The construction of pocket parks may not bring about a significant improvement in UGSS values but it could improve the living environment to some extent. Incremental improvements and changes in landscape design may not be fully captured by quantitative tools but these changes may be noticed by residents and have a positive effect on their feelings about the landscape. Therefore, the evaluation of UGS should include both quantitative and qualitative evaluation tools to measure indicators of improvements in UGS.

## Conclusion

Present UGS research tends to separate into street greenery and park greenery for the difference in measurement methods. In this paper, we combined street greenery and park greenery to which are frequently visited by residents without restriction in their daily lives to evaluate the UGS equity map. We did so by developing a measurement tool, the UGSS, to measure, evaluate, and score the differential UGS benefits in the Guangzhou Beltway region. The green benefit value of street greenery, SGS, was calculated by using the GVI. The green value benefit value of park greenery, PGS, was calculated based on the remote sensing data. The UGSS of the benefits enjoyed by residents was weighted and summed up, the service efficiency of UGS benefits were evaluated in combination with the population distribution.

We found that the overall street greenery in the study area is poor, the overall green benefit value of street greenery is low, and the distribution of street greenery in the study region is inequitable. The greenery benefit along the river is higher in the west of the study area and the middle and north of Haizhu District. The street benefit greenery in the east of Yuexiu District and Tianhe District is the lowest. However, the SGS values were restricted by the acquisition of panoramic images.

The PGS values in the study area were more evenly distributed than the SGS values; the high values may be linked to the large parks located. The benefit value of small parks is absorbed by the benefit value of large parks. However, the PGS values in the southwest corner of the study area are generally low. More than 10% of the study area was not included within the 1.5 km service areas of parks indicating an inequitable distribution of PGS values or park greenery.

The UGSS was combined with the green benefit of street greenery and park greenery and the score distribution is complementary. The UGSS values distribute unevenly, and the high values are distributed around the parks. The areas with lower PGS values in the southwest of the study area are supplemented by higher SGS values. The UGSS values of the central area of Guangzhou City, are low, which should be paid attention on. Combined with the population distribution, inequitable distribution is such that more than half the population in the study area does not enjoy good comprehensive UGS benefits. Furthermore, the UGSS values are lower in the areas with the highest population concentration.

Our goal in conducting this study was to gain a better understanding of the relationship between UGS and its benefits for area residents. In future studies on this subject, the combination of indices based on overlooking data and human visual data require further research. The weight between the two can be further distinguished according to the perception of the different population and different UGS benefit equity map for subdivision of residents such elders and young people. Urban construction and UGS planning should pay more attention to accessibility which is strongly related to environmental justice and equity. Planners should consider improvements in and equitable access to UGS and make high-quality public UGS available for all urban residents to enjoy.

## Supporting information

S1 Table (XLS)

S2 Table (XLSX)

S3 Table (XLS)

S4 Table (ZIP)
